# Reliable, versatile and remotely controlled instrumentation of an hectometric loop antenna using appropriate technologies

**DOI:** 10.1016/j.ohx.2023.e00463

**Published:** 2023-08-12

**Authors:** Clément Dezord, Gilles Micolau, Chahine Abbas, Arnaud Mesgouez, Elisabeth Pozzo Di Borgo

**Affiliations:** a UMR 1114 EMMAH, AU - INRAE, 301 rue Baruch de Spinoza, BP 21239, 84911 Avignon cedex 9, France; b UAR 3538 Laboratoire Souterrain à Bas Bruit (CNRS), 84400 Rustrel, France

**Keywords:** Huge loop antenna, Reliability, Harsh environment, Appropriate technology, Arduino–Raspberry association, Instrument control

## Abstract

The instrumentation of a giant loop antenna dedicated to environmental measurement, on a unique scientific site and intended to be open to the scientific community is presented. An open-source, low-cost electronic system has been designed, developed, and deployed in order to meet the need for reliability and flexibility imposed respectively by the harsh environment and the scientific objectives. The system’s architecture is based on the simultaneous association of five Arduino boards piloted together by a Raspberry Pi which also controls the measurement devices. The setup is therefore automated, pilotable, and remotely reprogrammable. Special attention was paid to its hardware and software reliability. These have been proven efficient over more than two years of operation. Several scientific conference publications have already proven the feasibility of the measurement principle (Dezord et al., 2021; Dezord et al., 2022; Dezord et al., 2022). This article gives previously unpublished details regarding the electronic setup.

## Graphical abstract

**Figure:**
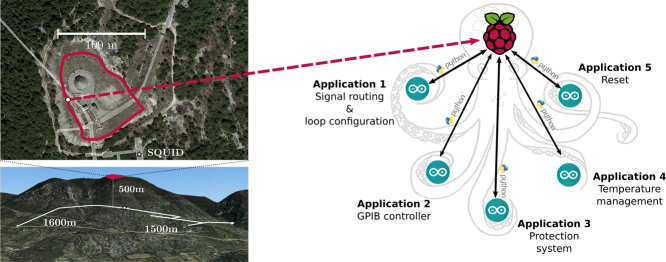

**Table:** 

Specifications table	

Hardware name	PentaPus – APIPy association
Subject area	Environmental sciences
Hardware type	Electrical engineering and computer science
Closest commercial analog	No commercial analog is available
Open source license	Creative Commons Attribution–NonCommercial–ShareAlike 4.0 International (CC BY–NC–SA 4.0)
Cost of hardware	∼1500 $
Source file repository	http://dx.doi.org/10.17632/z6hkzx433s.1

## 1 Hardware in context

### 1.1 Description of the experimental setup location

**Fig. 1:**
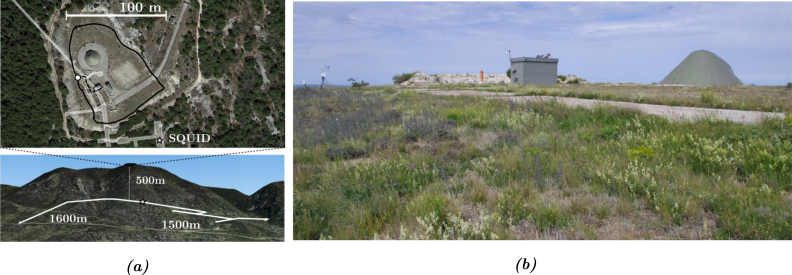
(a) Aerial (top) and southern (bottom) view picture of the Rustrel mountain. Black line represents the Vestale loop. White lines correspond to the underground galleries. The white star represents the position of the SQUID. The white circle represents the terminal box where the two ends of the Vestale loop meet. The dashed line shows the connection cables between the loop and the measuring instruments in the casemat. (b) Picture of the surface site of the LSBB where the Vestale loop is located.

A former command firing post of the French nuclear force, the Low Noise Underground Laboratory (named LSBB in French, [1]) located near Rustrel (Vaucluse — France), consists of an underground 4 km long gallery network, dug into the sold rock of the Grande Montagne (Fig. 1(a)). Covered at its deepest point by 500 m of solid rock, this environment offers a natural shield against radiation, flat access to the heart of the mountain, an extremely low mechanical and electromagnetic noise floor [2], and thermal stability at around +14°C throughout the year. Given these features, the original military construction was converted into an interdisciplinary underground laboratory. Here we now find unique hydrogeology experiments to follow the water load in the karstic massif [3], experiments to characterize muon detectors [4], experiments to study the construction possibilities for a gravitational wave detector [5], and even observations of transitional light events for the study of atmospheric phenomena [6]. Among all these experiments, a relative magnetometer with superconductor technology, named SQUID, was installed in the deepest galleries to profit from an environment shielded from electromagnetic frequencies higher than 40 Hz [7]. It is located in the former firing command post, and is encased in shielding metallic plates as well as being mechanically decoupled from the rock by a system of shock absorbers and cylinders.

About ten years ago, a magnetic excitation source was installed on the summit of the mountain, 500 m immediately above the SQUID, in order to follow the long-term response of the magnetometer to a perfectly controlled harmonic excitation under 40 Hz. The initial idea was to characterize, over time, any modifications in the hydric state of rock between the source and the underground SQUID.

The excitation source is a simple industrial cable laid on the ground and consisting of 12 copper wires of 1.5 mm^2^ insulated from each other and contained in an industrial plastic insulating sheath, 300 m long and connected to form a loop. The 12 conducting wires were initially connected together in order to form a giant 12-turn coil. The geometry of this coil was mainly constrained by the complex topography of the site. It therefore has an irregular shape, as shown in Fig. 1, with a characteristic average diameter of about 100 m and encompassing a total area of 5700 m^2^. This setup is called the Vestale loop.

### 1.2 Objectives of the experimental setup

The very special dimensions of this loop, its installation in an instrumented and multidisciplinary scientific environment, and its ability to continue working long-term provided researchers with the ideal conditions for its use as a giant sensor, at the scale of the mountainous massif. The final objective is to have a measuring instrument that is outstanding both by its size and by its location, and which will be open to different scientific communities whether they are present or not on the site. To our knowledge setups of this size, when they exist, do not possess simultaneously the three special features of the Vestale loop: immovable, open to use by different communities, remotely controlled and located in a densely instrumented environment [8], [9]. In this context, the dense multiphysics instrumentation of the LSBB presents enormous interest for the development of such a sensor. Its measurements could be correlated with a very large number of other measurements of different natures. One of the original features of this project lies in its approach. In the instrumentation domain, the nature and the precision required for the quantity measured usually dominate the specifications for the development of the sensor design. In this project, the starting point was the existence of a gigantic conductor loop, and we sought to extract the most information possible about its environment from it. The instrumental development therefore lay not in the optimization of the essentially unmodifiable sensor, but in that of the entire measurement system and of its piloting.

**Fig. 2:**
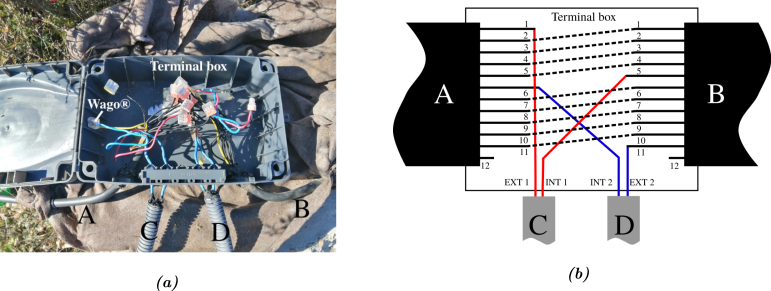
Picture (a) and connexion scheme (b) of the terminal box. A and B correspond to the two ends of the Vestale loop and C and D correspond to the extension cable routing the electrical signals between the loop and the instrument in the case at. In (b), solid lines correspond to the wires and dashed lines correspond to wago connections. The wire 1 from the end A roams the 300 m of the cable and comes back to the terminal box as the wire 1 of the end B. In the box, it is connected to the wire 2 from the end A and so on. At the end of the day, wires 1 to 5 form a 5 turn coil. In the same way, wires 6 to 11 form a 6 turns coil. Wire number 12 has no electrical continuity and is not connected to anything.

Four relevant functioning modes were identified. The first was the existing utilization mode of the Vestale loop as a magnetic field generator for the SQUID magnetometer. The second functioning mode consisted in using the loop as a magnetic sensor coupled to the mountain and to its immediate environment, rather like the sensors used in industry for non-destructive testing by eddy currents [10], [11], [12]. In this mode, the electrical impedance in the loop’s harmonic regime is measured over time. Its time evolution is characteristic of the surrounding media. The third identified functioning mode may be qualified as the passive mode. A voltmeter measures the voltage induced by inductive effect in the Vestale loop. Even if the number of turns is extremely low, the flux collection surface is gigantic (approximately $6.10^{4}$ m^2^). In these three modes, and in order to optimize the magnetic flux generated or received by the coil, all the wires in the loop must be connected in series. The fourth mode foreseen consists in using the loop as a giant fluxmeter [13], [14], [15]. To do so, some of the turn wires are connected to each other in series, forming a first coil, and the others, again connected to each other in series, form a second coil. Fig. 2(a) shows a photograph of the terminal box placed between the extremities of the 12 wire cable, as well as an electrical connection diagram.

The extremities of the Vestale loop cable are noted A and B. Wire number 12, in Fig. 2(b), cannot be used. Only the 11 wires numbered from 1 to 11 are functional. The wires 1 to 5, on the one hand, and 6 to 11, on the other, are connected in series to each other, using Wago®-type connecting terminals. Their ends (respectively denoted ext1/int1 and ext2/int2) are connected to two 35 m cables (respectively noted C and D in Fig. 2(b)) which enable the two coils thus formed to be independently addressed electrically. These two cables enable also the rooting of the electrical signals to the devices powering and controlling the coil, located in a bunker equipped with access to the LSBB electrical and IT networks.

We insist here on the essential scientific objectives of this experimental setup. They involve producing recordings in the long term (several months), whatever the functioning mode, of a selected electrical quantity.

### 1.3 Requirements for the facility

The instrumentation enabling the sensor to be controlled must deal with the different constraints related to the Vestale loop’s geographical position. It is located in an isolated area, subject to harsh climatic conditions (−10°C - +40°C, humidity, snow, etc.), unprotected from the wind, and at an altitude of 1000 m. Furthermore, access is difficult and the trip there takes a long time. Thus the first major point was to produce instrumentation which is automated, remotely pilotable, robust, and with reliable hardware and software.

Among the functions it offers, the developed system must not only retain its initial functionality as an excitation source for the SQUID, but also house new measuring instruments, while anticipating possible evolutions. To achieve this, a strategy of using suitably adapted technologies [16], [17], [18], based on inexpensive, open-source equipment, was adopted for the entire piloting and control/command part of the bench. In general, research prototypes must be highly flexible in their utilization and settings. The use of open-source technologies is completely suitable for the high versatility targeted in the system thus designed. Moreover, it contributes an important element to meet the great challenges of the laboratory equipment “debt” and of the durability of electronic systems. All too often, it has been seen that highly efficient measurement devices have to be replaced because their piloting systems depend on an obsolete proprietary OS whose updating is incompatible with the equipment [19], [20]. In this work, measuring instruments were chosen for their robustness and their recognized industrial quality.

### 1.4 Scope of this article

**Fig. 3:**
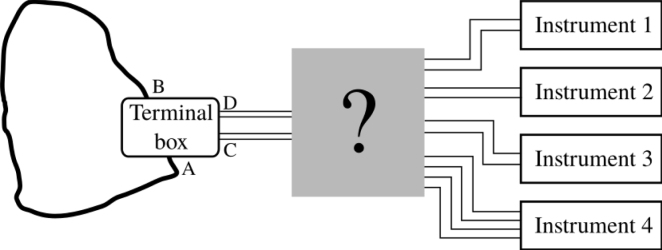
Explicit designation of the purpose of this article and its integration into the whole experimental setup, between the Vestale loop (black fat line) and the instruments. We will discuss the construction, the validation and the operation of the PentaPus which is here represented in gray.

At present, remote commands mean the Vestale loop can be connected to two measurement devices, an LCRmeter and a precision multimeter. They respectively allow for the monitoring of the loop’s electrical impedance and its induced electrical voltage over a long period. As far as we know, none of the very few comparable installations are neither able to perform such a long term monitoring or remotely controlled. To do so, the Vestale loop is linked to a remotely pilotable junction box, offering a choice of the type of measurement. Once launched, the measuring is completely automated. The box is linked to a controller accessible via network, and that also pilots the measurement devices. The box can manage two other devices, corresponding to active magnetic source functioning mode and to an as-yet undeveloped fluxmeter mode. The feasibility and measurement appropriateness demonstrations have been presented in [21], [22], [23]. However, the setup architecture, its detailed deployment, and its functioning were not described there. The objective of this article is to share informations related to the design and the construction of the entire system of control/command and piloting instrument. This device is subsequently named *PentaPus*. Fig. 3 shows its integration between the instruments and the Vestale loop and thus the overall architecture of the experimental setup. On one side, this device has ten terminals leading to the different instruments. On the other side, it has four terminals to the Vestale loop. This device, the PentaPus, containing a set of relays, power supply systems, and different controllers, is the main subject of this article.

The system was built under strict lockdown conditions which prevailed during the COVID-19 pandemic in 2020. This added a further constraint that had to be integrated in the prototype development work. In particular, a posteriori, this situation enabled clear confirmation of the appropriateness of the choice of suitably adapted, open-source technology for the development of a truly “home-made” solution.

## 2 Hardware description

This section is organized in eight sub-sections. The first gives a general presentation of the PentaPus architecture, while defining the APIPy association and specifying the interest of its multiple utilization. The following five sub-sections explain the functioning principles, and the roles of the APIPy. Then, a sub-section precises the specifications of the controlled instruments that are not included in the PentaPus. Finally, a summary gives a more detailed presentation of the PentaPus architecture and its integration in the whole experimental setup.

### 2.1 General architecture of the PentaPus based on a generic and modular solution, the APIPy

The central system controller is a Raspberry Pi 3, functioning under the Linux Raspbian OS. Open-source, reliable, maintained, and free of charge, this OS provides to the nano-computer a highly efficient network communication protocols in the command line. It is therefore possible to carry out complex operations remotely, without needing very large network or graphic resources. Based on a System on Chip (SoC) BCM2837 [24], the Raspberry Pi 3 has an Ethernet port, four USB ports, and an interface for logic inputs and outputs (GPIO — General Purpose Input/Output). Unfortunately, these GPIOs are digital ports directly connected to the SoC. This direct connection to the processor makes the nano-computer extremely sensitive to short-circuits, and consequently to prototyping. Furthermore, their low number limits the possibilities for exploitation and therefore the level of versatility and flexibility that can be expected.

**Fig. 4:**
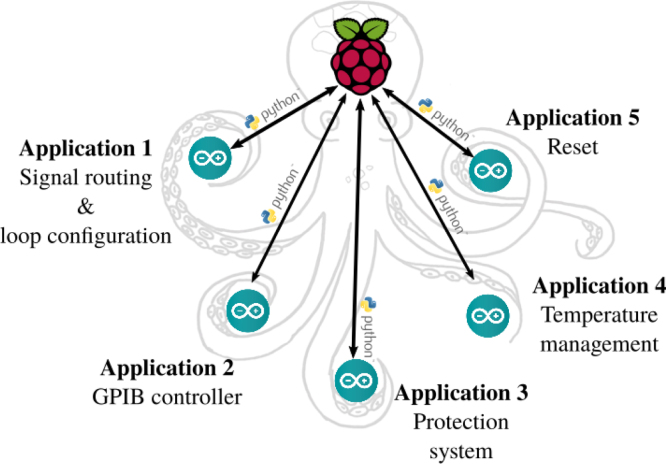
Architecture of the PentaPus. Five arduinos – corresponding to five applications – are controlled by a Raspberry PI via Python scripts. This architecture relies on five APIPy associations sharing their Raspberry Pi.

To get around these limitations, we chose to associate the Raspberry Pi 3 with an electronic prototyping board that is also open source, inexpensive, and very popular: the Arduino *Uno* [25]. This board, that operates the ATMega328P micro-controller from Microchip® [26], is easy to physically interface because of its USB interface and its standardized GPIO ports. It also has protected multi-purpose functionalities on-board, ideal for prototyping. These features make it compatible with modular extensions available on the market or constructed “in-house”. Fig. 4 gives a functional diagram of the control/command architecture. At present, the Raspberry is connected to five Arduinos, each carrying out a functionality. This architecture’s similarity to that of an octopus (1 central brain and brains in the tentacles) inspired the name of the system, PentaPus.

The software interfacing of the Arduinos is operated by Raspberry Pi through the execution of IT programs. The Python language, which is widely used by computer programmers, was chosen for these [27]. The user becomes familiar with it quickly, and it has a wide range of libraries, notably the “pySerial” library that enables communication with the Arduinos. In our case, the principle of this Arduino – RaspberryPI – Python association is based on an Arduino routine with a permanent listening watch on its USB port. The Raspberry will thus send the user’s instructions via the execution of a Python script (see Section 6).

The non-stop association between the Raspberry and the Arduino, ensured by a USB port and piloted by Python scripts, is referred to here as APIPy (for Arduino-PI-Python). It can be deployed to carry out numerous functionalities. In practice, a USB hub enables an increase in the number of Arduinos connected. It also ensures that they have a reliable, correctly dimensioned power source.

### 2.2 APIPy 1: configuration of the loop

**Fig. 5:**
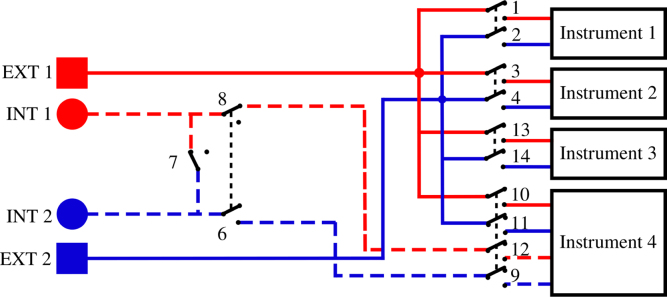
Principle description of the configuration and of the signal routing. Blue lines correspond to the 5-turns loop. Red lines correspond to the 6-turns loop. Solid lines correspond to the terminals of the 11-turns loop of configuration (1). Dashed blue or red lines correspond either to the connection in series (configuration (1)) or to the two other terminals in configuration (2). Black dashed lines between relays indicate an electrical lock. The relay 7, 8 and 9 configure the loop. Especially, the relay 7 join the two sets of loop. To configure the loop, there is an electrical lock between relay 7 and relay 8 and 9 : when the relay 7 is close, the relays 8 and 9 are open and the loop is in configuration (1); while when the relay 7 is open, the relays 8 and 9 are close and the loop is in configuration (2). There is an electrical lock between the 1–2 relay set, 3–4 relay set, 13–14 relay set and 9–12 relay set; the relay name correspond to the following Fig. 8, Fig. 9, Fig. 10. (For interpretation of the references to color in this figure legend, the reader is referred to the web version of this article.)

The connection of the different strands of the Vestale loop’s cable are shown in Fig. 2. They make it possible to consider two different configurations : (1) a single 11-turn loop in series, or (2) two separate loops consisting respectively of 5 turns in series and of 6 turns in series. The main objective of the PentaPus is to direct an electrical signal coming from the two extremities of configuration (1) — or from the four extremities of configuration (2) — towards the selected measuring instrument. Selecting the configuration and routing the signal were managed by the first APIPy deployment. Electromechanical relays were used to carry out these two functionalities. The relays have the advantage of being robust and affordable, and constitute a clear separation between the digital control/command system and the analog measurement channel. The APIPy module is used to manage the relays. In order to change from configuration (1) to configuration (2), three relays are necessary (Fig. 5). In configuration (1), the signal is routed towards three different instruments. For each instrument, two relays – one for each end – are necessary. In configuration (2), one relay is necessary per end, i.e. a total of four. In our case, 13 relays were necessary to configure the loop and direct the signal. An electronic board with 16 modular 10A-relays [28], compatible with an Arduino and classically used in the prototyping field [29] was installed. Hereafter it is called RB16a.

### 2.3 APIPy 2: GPIB converter

One of the essential functionalities of PentaPus is the piloting of measuring instruments. This piloting role is ensured by the Raspberry duly connected to each measurement device on the bench. At present, one of them is GPIB-interfaced. Although obsolete, this protocol is a robust standard and still widely found [30]. In particular, it enables the cascading of several devices on the same GPIB bus. This offers the possibility of adding devices in the future, and thus is completely compatible with the versatility and modularity sought.

A USB/GPIB converter must be used to connect the Raspberry to the controlled measuring instrument. Converter products currently on the market are not compatible with Linux distributions for Raspberry. We therefore designed a USB/GPIB controller working with the APIPy module.

This design was based on the open source Agipibi project [31] to build a USB/GPIB controller with the APIPy module. This project offers source codes and the connection plan to create an inexpensive USB-GPIB interface with an Arduino *Mega*. In this work, slight modifications have been made to the electronics of this project to make it suitable to an Arduino *Uno*.

### 2.4 APIPy 3: protection against lightning

The location of the Vestale loop on top of a mountain exposes the equipment to lightning strikes, particularly via the loop itself. Beyond the surge arrestors already installed (see Section 5.3), it is wise to plan for a standby state for the entire system when it is not functioning.

**Fig. 6:**
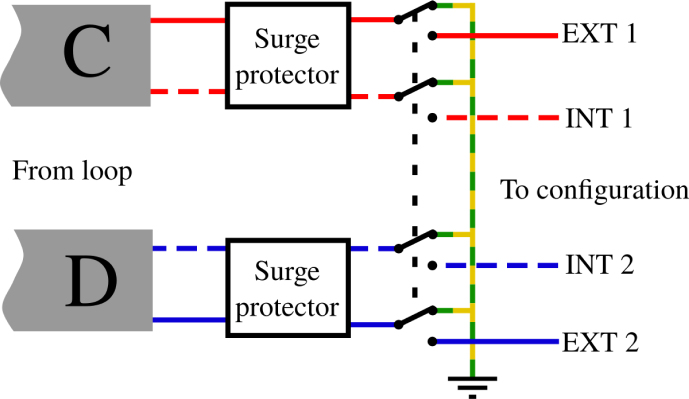
Earthing principle of the Vestale loop. C and D letters correspond to the extensions coming from the terminal box.

When the measurements have been stopped, the measurement devices are completely disconnected to isolate them from the Vestale loop. The ends of the loop are linked together and earthed, thereby offering additional protection against lightning damage. The APIPy 3 module pilots the four relays necessary for the ends of the loop (Fig. 6). A four 10A-relays board is used, hereafter called RB4a. This protection system is placed as close as possible to the extension cables from the loop before the APIPy 1. It is also physically distanced from the rest of the measurement bench’s electronic systems.

### 2.5 APIPy 4: temperature regulation

The temperature within the bunker ranges from −10°C to +40°C. To keep the Raspberry and the measuring instruments at suitable temperatures for them to be used, they are placed in a thermally controlled enclosure where the temperature is maintained at between +25°C and +28°C. A tropicalized household refrigerator, without a freezer compartment (to avoid the presence of liquid water should it break down), and with no speed regulator was modified to perform this function. The main modification was the removal of the electromechanical thermostat and its replacement by a relay and two temperature sensors. Finally, a household blower heater, also piloted by a relay, was set up inside. An ON–OFF control triggers the heater when the internal temperature drops below +25°C, and starts up the refrigerator compressor when it goes above +28°C.

Two relays are necessary for these operations. A four 10A-relays board is connected to the APIPy 4, hereafter called RB4b.

### 2.6 APIPy 5: instrument reset and enhanced reliability

In order to ensure the most comprehensive remote management of the measurement bench, it is necessary to be able to switch off the measuring instruments (which may potentially influence each other) as well as the APIPy. To do this, relays were installed on the power supplies of each of them. Two power supply voltages are involved, as the measurement instruments have 230 V AC and the APIPy receive 5 V DC via their USB ports. As the Arduino’s power is via the USB hub, their power supply relays are placed directly on the USB hub switches. For the measuring instruments, the relays are placed directly on their 230 V power supplies via an extension. Finally, additional relays are necessary to shunt a resistor used to make the Arduino functioning more reliable. This point will be discussed in Appendix A.1 [32]. A total of eleven relays is necessary for this functionality. The APIPy 5 is therefore associated with a sixteen 10A-relays board, hereafter called RB16b.

Moreover, the startup times for the Arduinos, the relay boards, and the measuring instruments are different.

### 2.7 Existing measurement instruments

The use of intermediary technologies is adapted to remote piloting and control. In particular, it enables financial resources to be concentrated on the purchase of industrial precision measurement instruments known for their good performance and their reliability. In this work, impedance measurements are performed by the LCRmeter E4980 A from Keysight [33]. It enables complex electrical impedance measurements to be carried out over a frequency range of 20 Hz to 300 kHz, with a 1 V voltage and a current level of approximately 10 mA. It is connected via GPIB to the Raspberry via APIPy 2. The induced voltage measurement is performed by the DAQ6510 precision multimeter from Keithley [34]. This instrument combines two distinct functionalities: a precision multimeter mode and an 80 channel acquisition board mode. Here it is used exclusively in multimeter mode, configured as a voltmeter. This instrument can be interfaced via USB and is directly plugged into the USB Hub. Finally, the device has a 5 million sample buffer memory, available at the end of the measurement.

### 2.8 Summary

**Fig. 7:**
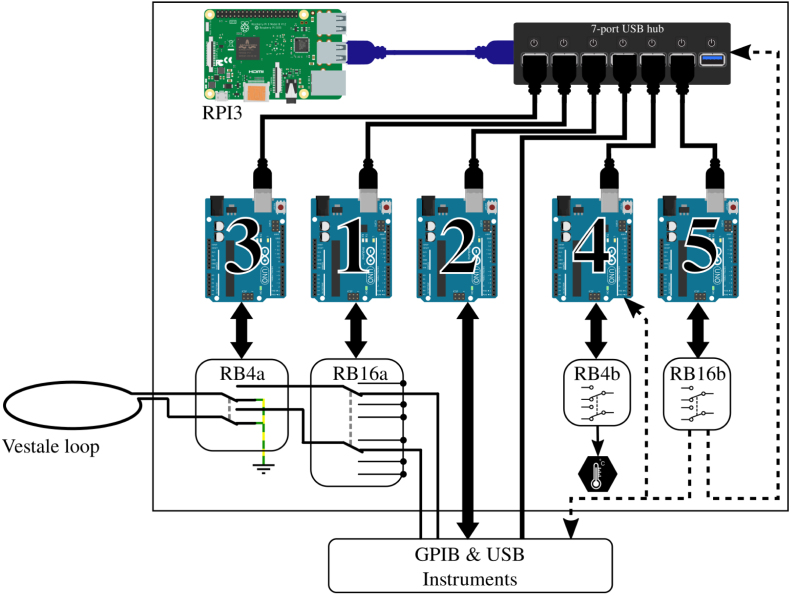
Pentapus detailed architecture and integration in the whole experimental setup, between the Vestale loop and the measuring instruments. Main hardware devices controlled by arduinos of each APIPy are precised: relay boards, GPIB controllers, and temperature control system.

Fig. 7 shows a detailed diagram of the PentaPus and of its environment. The different functionalities implemented by the five APIPys use relays grouped on different relay boards. The relays have been grouped on boards corresponding to a single functionality and therefore associated with a single Arduino, without seeking to optimize the number of relay boards.

APIPy 1 is associated with the configuration of the loops and the signal routing. It pilots the RB16a relay board. APIPy 2 is associated with the GPIB controller. APIPy 3 is associated with the earthing. It pilots the RB4a relay board. APIPy 4 is for the temperature regulation. It pilots the RB4b relay board. Finally, APIPy 5 is the one that reinitializes and makes the measurement bench reliable. It pilots the RB16b relay board.

## 3 Design files summary

All Design files below are located in 10.17632/z6hkzx433s.1 [32]. These files are under CC BY-NC-SA 4.0 open source license and they are organized by categories. The “RST” python scripts allow advanced functionalities of the APIPy, as reboot, and control the measuring instruments. The “RTG” python scripts allow to rout the signal. The “DAQ” and “LCRmeter” files allow to set and control the automation of the measuring instruments. The data files are examples of different measurements carried out with the instruments and the refrigerator.

**Table:** 

Design filename	File type	Design filename	File type
USB-GPIB	Electronic PCB file	RST_USB7ON.py	Python script
agipibi.py	Python script	RST_RoffAPIPy4.py	Python script
arduino.py	Python script	RST_RoffAPIPy5.py	Python script
DAQScript.py	Python script	RST_DAQ6510OFF.py	Python script
DAQSettings.txt	Text file	RST_LCRmeterOFF.py	Python script
EarthingConnection.py	Python script	RST_USB7OFF.py	Python script
EarthingDisconnection.py	Python script	RTG_6Series5Series.py	Python script
LCRmeterScript.py	Python script	RTG_11Series.py	Python script
LCRmeterSettings.txt	Text file	RTG_Connection2wires.py	Python script
RST_RebootAPIPy4.py	Python script	RTG_Connection4wires.py	Python script
RST_RebootAPIPy2.py	Python script	RTG_ConnectionDAQ6510.py	Python script
RST_RebootAPIPy1.py	Python script	RTG_ConnectionLCRmeter.py	Python script
RST_RebootAPIPy3.py	Python script	TEMP_Recording.py	Python script
RST_RebootAPIPy4.py	Python script	20230117_DS18b20.dat	Data file
RST_RonAPIPy4.py	Python script	20220504_LCRmeterE4980A.dat	Data file
RST_RonAPIPy5.py	Python script	20230117_DAQ6510.dat	Data file
RST_DAQ6510ON.py	Python script	Appendix	Pdf file
RST_LCRmeterON.py	Python script	–	–

## 4 Bill of materials summary

This BOM refer to the Section 5.6 and in Appendix A.14 [32]. In the following, the list will mention only the material related to the functionalities. The consumables such as wires, basic electronics components, prototyping boards, hardware are given as an indication.

### 4.1 PentaPus system

**Table:** 

Component	Number	Cost per unit	Total cost	Source of	Material
		[USD]	[USD]	materials	type
Raspberry Pi 3	1	78.11	78.11	conrad.fr	Electronics
RPI3 power supply	1	14.71	14.71	conrad.fr	Electronics
Ethernet cable	1	6.22	6.22	conrad.fr	Electronics
2-meter USB cable	5	7.35	36.75	conrad.fr	Electronics
USB hub	1	100.76	100.76	conrad.fr	Electronics
Arduino Uno Rev. 3	5	27.29	136.45	rs-online.com	Electronics
Resistor 33 Ω±1%	5	0.0036	0.018	rs-online.com	Electronics

### 4.2 APIPy 1: configuration of the loop

**Table:** 

Component	Number	Cost per unit	Total cost	Source of	Material
		[USD]	[USD]	materials	type
Arduino Uno Rev. 3	1	27.29	27.29	rs-online.com	Electronics
Prototyping board	1	5.79	5.79	rs-online.com	Electronics
USB cable	1	7.35	7.35	conrad.fr	Electronics
Resistor 33 Ω±1%	1	0.0036	0.0036	rs-online.com	Electronics
16-relay module	1	30.58	30.58	rs-online.com	Electronics
BS170 transistor	4	0.24	0.96	conrad.fr	Electronics
Resistor 2.7 kΩ±1%	4	0.0036	0.0144	rs-online.com	Electronics
Resistor 12 kΩ±1%	4	0.0036	0.0144	rs-online.com	Electronics
Capacitor 100 nF	4	0.12	0.48	rs-online.com	Electronics

### 4.3 APIPy 2: GPIB converter

**Table:** 

Component	Number	Cost per unit	Total cost	Source of	Material
		[USD]	[USD]	materials	type
Prototyping board	1	5.79	5.79	rs-online.com	Electronics
Arduino Uno Rev. 3	1	27.29	27.29	rs-online.com	Electronics
Resistor 33 Ω±1%	1	0.0036	0.0036	rs-online.com	Electronics
Standard LED	1	0.54	0.54	conrad.fr	Electronics
USB cable	1	7.35	7.35	conrad.fr	Electronics
GPIB cable	1	96.48	96.48	conrad.fr	Electronics
Instrumentation box	1	17.61	17.61	rs-online.com	Electronics

### 4.4 APIPy 3: protection against lightning

**Table:** 

Component	Number	Cost per unit	Total cost	Source of	Material
		[USD]	[USD]	materials	type
Arduino Uno Rev. 3	1	27.29	27.29	rs-online.com	Electronics
4-relay module	1	11.07	11.07	rs-online.com	Electronics
Surge arrestor	2	12.39	24.78	castorama.fr	Appliance
Resistor 2.7 kΩ±1%	1	0.0036	0.0036	rs-online.com	Electronics
Resistor 12 kΩ±1%	1	0.0036	0.0036	rs-online.com	Electronics
Capacitor 100 nF	1	0.12	0.12	rs-online.com	Electronics
BS170 transistor	1	0.24	0.24	conrad.fr	Electronics

### 4.5 APIPy 4: temperature control

**Table:** 

Component	Number	Cost per unit	Total cost	Source of	Material
		[USD]	[USD]	materials	type
Refrigerator 240 L single door	1	225.85	225.85	electromenager-compare.com	Appliance
Arduino Uno Rev. 3	1	27.29	27.29	rs-online.com	Electronics
4-relay module	1	11.07	11.07	rs-online.com	Electronics
DS18b20 sensor	2	16.76	33.52	conrad.fr	Electronics
Resistor 33 Ω±1%	1	0.0036	0.0036	rs-online.com	Electronics
Resistor 2.7 kΩ±1%	2	0.0036	0.0072	rs-online.com	Electronics
Resistor 12 kΩ±1%	2	0.0036	0.0072	rs-online.com	Electronics
Capacitor 100 nF	2	0.12	0.24	rs-online.com	Electronics
BS170 transistor	2	0.24	0.48	conrad.fr	Electronics

### 4.6 APIPy 5: instrument reset

**Table:** 

Component	Number	Cost per unit	Total cost	Source of	Material
		[USD]	[USD]	materials	type
Arduino Uno Rev. 3	1	27.29	27.29	rs-online.com	Electronics
16-relay module	1	30.58	30.58	rs-online.com	Electronics
Resistor 33 Ω±1%	1	0.0036	0.0036	rs-online.com	Electronics
74HC14 NOT gates	3	0.38	1.14	conrad.fr	Electronics
1N4148 diode	1	0.16	0.16	conrad.fr	Electronics
Resistor 2.7 kΩ±1%	2	0.0036	0.0072	rs-online.com	Electronics
Resistor 12 kΩ±1%	2	0.0036	0.0072	rs-online.com	Electronics
Capacitor 100 nF	1	0.12	0.12	rs-online.com	Electronics
Capacitor 2.2 mF	1	2.4	2.4	rs-online.com	Electronics
BS170 transistor	2	0.24	0.48	conrad.fr	Electronics
Delaying relay	1	3.15	3.15	rs-online.com	Electronics
Terminal	2	0.71	1.42	conrad.fr	Electronics

### 4.7 Power supply to the measurement bench

**Table:** 

Component	Number	Cost per unit	Total cost	Source of	Material
		[USD]	[USD]	materials	type
UPS Eaton 5P850I 850 VA	1	392.16	392.16	conrad.fr	Appliance
Domestic multisocket	2	19.82	39.64	castorama.fr	Electronics
3-meter power cord	2	12.26	24.52	conrad.fr	Electronics
Derivation box	5	7.85	39.25	conrad.fr	Electronics

## 5 Build instructions

Here are given the necessary instructions for the Pentapus construction, whose modular sub-systems were designed independently from each other. Once completed, the last section presents an overview of the PentaPus and its integration into the experimental setup, between the loop and the measuring instruments. The specific designs providing electric reliability are presented directly in Appendix A located in the repository [32]. Particularly, a hardware solution is proposed to avoid the well-known intempestive reset of Arduinos under Linux system.

### 5.1 APIPy 1: configuration of the loop

**Fig. 8:**
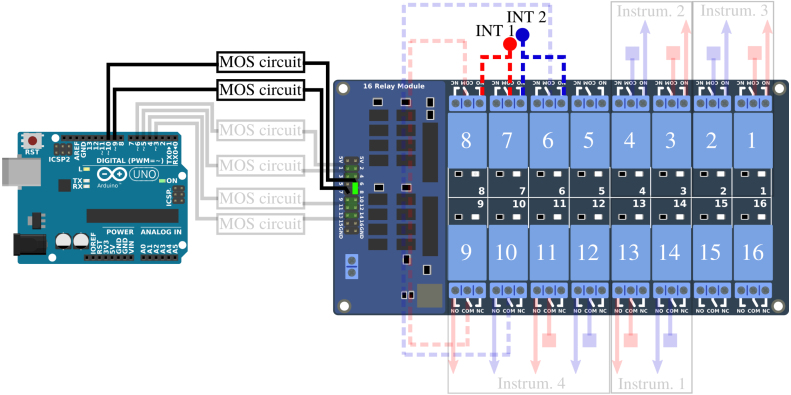
Part 1/3. Wiring scheme of the loop configuration functionality. (For interpretation of the references to color in this figure legend, the reader is referred to the web version of this article.)

**Fig. 9:**
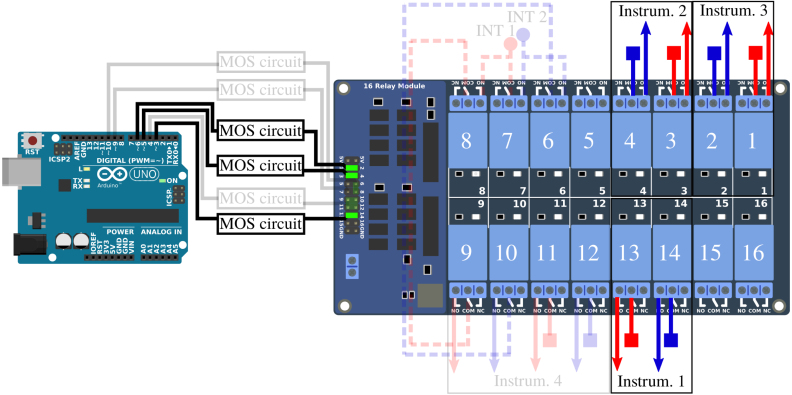
Part 2/3. Wiring scheme of the routing towards a 2-channels instrument.

**Fig. 10:**
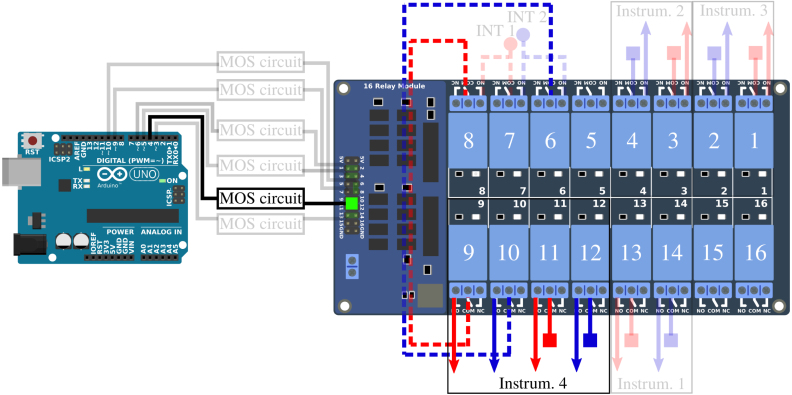
Part 3/3. Wiring scheme of the routing towards a 4-channels instrument.

This sub-section presents the two functionalities of the RB16a piloted by the APIPy 1 : the configuration of the two sub-loops and the signal routing towards the four different instruments (Fig. 5). Three of these instruments must be connected to the exterior ends of the two sub-loops (solid lines, square symbol in Fig. 8, Fig. 9). These two ends are therefore derived to then be connected independently to the different instruments. The last instrument is connected to the four ends of the two sub loops (solid lines, square symbol and dotted lines, round symbol on Fig. 10). For clarity, this sub-section is divided into three instructions. The first concerns the loop configuration. The second concerns the signal routing towards the two-channel instruments and the third the signal routing towards the four-channel instrument. Appendix A.4 [32] describes how the ends of the loops were split between the different measuring instruments.

*Configuration of the loop –* The two interior terminals (broken line, round symbol) arrive from the MALT board and are routed towards relays 6, 7, and 8. When relay 7 is closed, relays 6 and 8 are open. The loop is in the configuration with 5 and 6 independent turns. When relay 7 is open, relays 6 and 8 are closed and the loop is in the configuration with 11 turns in series. Relays 6 and 8 are always activated simultaneously.

The interior terminal of the 6 turn sub-loop (broken line, round blue symbols) must be connected to the NO contact of the relay 7. The interior terminal of the 5 turn sub-loop (broken line, round red symbols) must be connected to the common contact of the relay 7. For these two terminals coming from RB4a, the wiring will be specified in Appendix A.3 [32]. The relay 6 NO contact must be connected to the relay 7 NO contact. The relay 8 NO contact must be connected to the common contact of the relay 7. Each time, a rigid $1.5\;{\mathrm{mm}}^{2}$ section cable was used. The signal coming from the two independent sub-loops will transit by the common contact of relays 6 and 8. Their wiring is described in Appendix A.3 [32]. Fig. 8 depicts the electrical diagram corresponding to this first set of instructions.

*Routing towards a 2-channels instrument –* After their junction (see Appendix A.4 [32]), the two exterior terminals (solid line, square symbol) are linked to three pairs of relays to be routed towards the first three instruments. Relays 1 and 2 route the signal towards the LRCmeter, while relays 3 and 4 route it towards the DAQ and relays 13 and 14 to the power supply. Each time these pairs of relays are activated simultaneously.

The different loop’s terminals are made available by their connection to the common contact of the relays 1, 2, 3, 4, 13, and 14. The NO contact of these six relays enables their connection to the instruments. A rigid 1.5 mm^2^ section cable is used between the junction box and the relays, and between the relays and the terminals.

*Routing towards a 4-channels instrument –* The last instrument is connected to four terminals. The two exterior terminals (solid line, square symbol) are connected to this instrument by relays 11 and 12 while the two interior (broken line, round symbol) are connected by relays 9 and 10. These four relays are activated simultaneously.

The exterior terminals are made available by their connection to the common contact of relays 11 and 12. The interior terminals, connected to the common contact of relays 6 and 8 when the loop is configured in two sub-loops, are made available by their connection to the common contact of relays 9 and 10. The NO contact of these four relays enables their connection to the instrument. Here again, a rigid 1.5 mm^2^ section cable is used between the junction box and the relays, and between the relays and the terminals.

Appendices A.3 and A.4 [32] summarize all the instructions corresponding respectively to the driving of the RB16a by the APIPy 1 and to the connection of the relay contacts. Instructions regarding the power supply of the RB16a are given in Appendix A.12 [32].

### 5.2 APIPy 2: GPIB converter

**Fig. 11:**
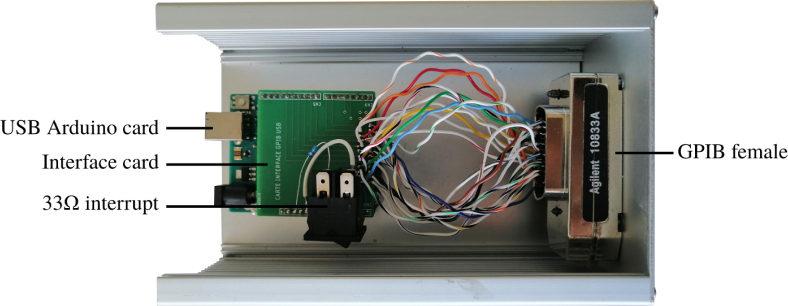
Internal view of the home-made GPIB controller. A custom-made interface board is mounted on the Arduino to ensure reliable soldering of the wiring between the GPIO pins and the GPIB socket’s pins.

The controller is housed in a robust casing, within which the Arduino and a GPIB socket are attached. The walls of the box were pierced to allow access to the Arduino’s USB slot and to the GPIB socket (Fig. 11). Thus, a complete system is obtained, comparable to commercially-available devices [35], [36]. A robust mechanical support of the Arduino is provided by the use of standoffs.

The GPIO ports of the Arduino are connected to different pins of the GPIB socket via a specially designed electronic board, whose plan is given in Appendix A.5 [32]. The main role of this board is to facilitate the wiring between the Arduino and the terminal of the GPIB socket. It is absolutely essential to install a 33 Ω resistor between the “+5V” pin and the “Reset” pin on the Arduino of the APIPy 2. The connection board between the Arduino ports and the pins on the GPIB connector is shown in Appendix A.5 [32] too.

### 5.3 APIPy 3: Protection against lightning

To prevent any damage that could be caused by a lightning strike, the entire setup is protected by surge arrestors and by an earthing system. If the two hardware solutions are presented here, only the earthing system is based on the APIPy architecture.

*Surge arrestor –* The ends of the loop are equipped with 2 electrical sockets for classical power. Two household-type surge arrestors are plugged directly into these sockets. While keeping their outer structures, these surge arrestors were modified to limit any influence they might have had on the signals (see Appendix A.6 [32]).

In our case, we took apart the surge arrestors to remove all the components likely to influence the loop signals. The first change concerned the removal of the light indicating the device’s active protection. The second and third changes involved respectively the removal of the switch light and of its shunt. After these modifications, only the thermal fuse and its varistor remain between the two terminals of the two loops.

**Fig. 12:**
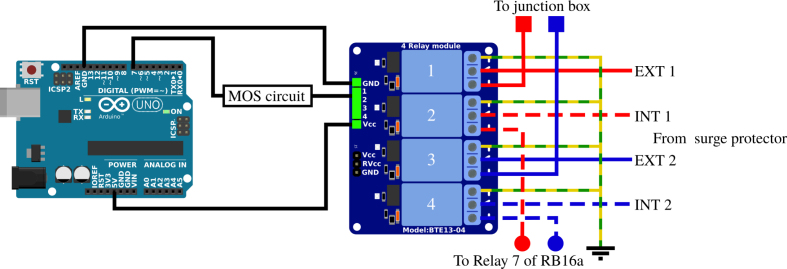
Wiring map of APIPy 3 and the RB4a.

*Earthing –* The four terminals of the loop each arrive on the COM contact of the RB4a relay (Fig. 12). An appropriately sized earthing cable is connected to the NC terminals of the four relays (Appendix A.7 [32]). The two exterior ends are on the relay 1 and 4 NO contacts of the RB4a and go towards the terminal box, while the 2 interior ends are on the relay 2 and 3 NO contacts of the RB4a and go towards relay 7 on the RB16a. All the relays are activated simultaneously.

Appendix A.7 [32] summarize all the instructions corresponding respectively to the driving of the RB4a by the APIPy 3 and to the connection of the relay contacts. Thus connected, when all the relays are inactive, the four loop ends are short-circuited and earthed. Power for the RB4a board is supplied by the APIPy 3.

### 5.4 APIPy 4: Temperature regulation

**Fig. 13:**
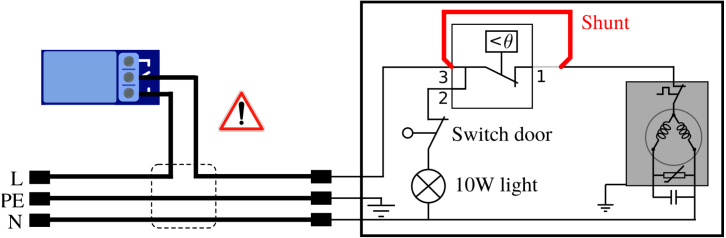
Electrical scheme of a domestic refrigerator. The red line shows the required modification to shunt the thermostat. The dark gray rectangle on the right corresponds to the refrigerator compressor. The warning symbol mentions the presence of hazardous voltage in this wiring part. (For interpretation of the references to color in this figure legend, the reader is referred to the web version of this article.)

**Fig. 14:**
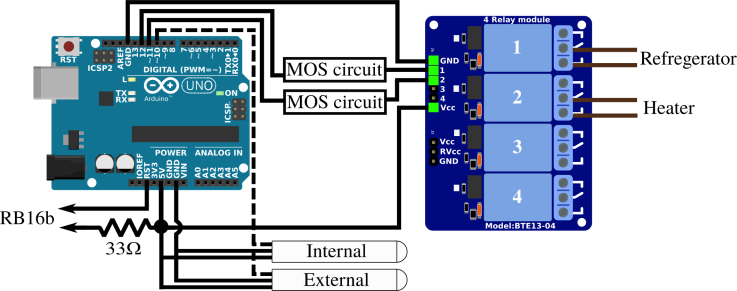
Wiring map of APIPy 4 and of the RB4b. The RST pin of the Arduino and the end of the 33 Ω resistor go to the RB16b (Appendix A.1 [32]). Here the refrigerator power line correspond to the L line of the Fig. 13.

The initial refrigerator temperature regulation system was based on the principle of a dry contact thermostat, activating the compressor in “ON-OFF control”. To implement a customized regulation, this thermostat was replaced by a relay and a digital temperature probe (Fig. 13, Fig. 14). To do so, the refrigerator is modified and the power lines are wired on the APIPy 4. The power supply to the refrigerator and to the heater were also modified in order to be piloted by the RB4b relays. These modifications are presented in the last part of Appendix A.12 [32].

*Modification of the refrigerator –* The thermostat shunt consisted first in dismantling the thermostat unit located within the appliance. Identification of the 230 V feed and the compressor feed is required. To do so, start by unplugging the fridge. Then remove the thermostat and push the door interrupt in order to disconnect the light circuit (wire 2 of the thermostat in Fig. 13) from both the neutral (door interrupt) and the phase (230 V feed that is wire 3 of the thermostat in Fig. 13). Using a digital multimeter in continuity test mode, find the only wire that has no electrical continuity with neither the phase nor the neutral. The two other wires correspond to position 1 and 3 of the thermostat in Fig. 13. Connect them in order to shunt the thermostat.

Installation of equipment inside the refrigerator required at least one hole to be made in the lateral wall or in the door of the appliance. Here, attention must be paid to the presence of capillary pipes in the wall for the cooling liquid. They can be detected when the refrigerator is switched on, with the door open, by the presence of condensation on the inner wall.

*Wiring APIPy 4 –* The APIPy 4 pilots power supply to the refrigerator and to the blower heater independently through relays 1 and 2 of the RB4b. The RB4b board power is supplied by the APIPy 4 (see Appendix A.8 [32]). The temperature regulation managed by the APIPy 4 requires the acquisition of the temperature inside the refrigerator, using an encapsulated DS18b20 probes [37]. A second identical probe is also used to monitor the temperature outside the appliance. These probes use a OneWire bus and are supplied with +5V by the APIPy 4. Instructions regarding all these connections, including the switchable 33 Ω resistor, are depicted in Fig. 14 and in Appendix A.8 [32].

### 5.5 APIPy 5: instrument reset and enhanced reliability

To be able to control the measurement instrument and APIPy, a function based on an Arduino and RB16b is carried out. Four instructions are given below to build this functionality.

**Fig. 15:**
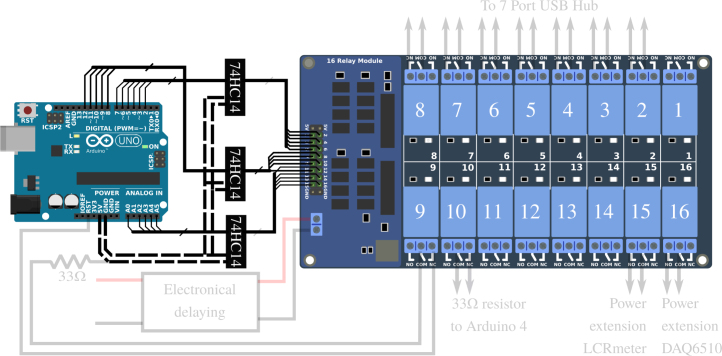
Part 1/3. Wiring map of the control of the relay board RB16b of the APIPy 5.

*RB16b command circuit –* Each of the relays of the RB16b is piloted independently. To avoid multiplying unnecessarily the number of amplifier/inverter units presented in Appendix A.2 [32], we chose (solely for this configuration) to use integrated logic gates (Fig. 15). The component used is a 74HC14 with seven NO gates, which enable the signals to be reversed. To keep the possibility of piloting the five relays available on the RB16b, we used three of these integrated logic circuits. They also amplify the signal. They are powered with +5V via the APIPy 5 Arduino.

The first 74HC14 pilots relays 1 to 6 from the GPIO 2 to 7 of the Arduino. The second 74HC14 pilots relays 7 to 11 from the GPIO 8 to 12 of the Arduino, while the third 74HC14 pilots relays 13 to 16 from the GPIO A0 to A4 of the Arduino. The Ground and Vcc ports of the three 74HC14 are electrically contacted 3 by 3 and connected respectively to the GND terminal and to the +5V terminal of the APIPy 5. The three 74HC14 are placed on the prototyping board. All the informations regarding the corresponding wiring are depicted in Fig. 15 and explicited in Appendix A.9 [32].

**Fig. 16:**
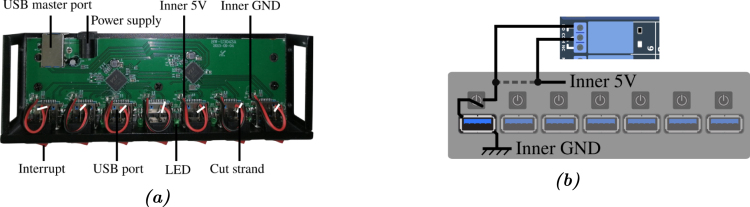
(a) Top view of the opened 7-port USB hub. (b) Electric schematic of one modified switch. The inner +5V wire can be to cut (dotted line). The first end is soldered on the inner +5V (black circle). The second end is soldered directly on the interrupt (black circle). The others ends of the new wire is wired in accordance with Appendix A.10.

*Modification of the hub –* To keep the functionality of each manual switch, they were connected in series on the RB16b relays (Fig. 16). To do so for each of the switches, the wires initially connected to the internal +5V power supply of the hub were cut, then each connected to the NC contact of respective relay. The common contact of these relays was then connected to the internal +5V power supply of the hub.

*Connection of the relay contacts –* The RB16b board pilots the power supply to the APIPy, to the measurement instruments, and to the R33 of APIPy 4 and 5. The hub connecting the Arduinos to the Raspberry is also used to supply them. We chose a model with independent switches, which we modified to set up a relay in series with each interrupt. Relays 2 to 8 of the RB16b are used for the 7 interrupt of the hub. The two pilotable 33 Ω resistors ensuring the reliability of APIPy 4 and 5 were mounted on relays 9 and 10 to enable the corresponding APIPy to be reprogrammed. Finally, relays 15 and 16 respectively enable the power supply to the LCRmeter and the DAQ to be piloted. The contact connections for relays 15 and 16 corresponding to the power supply to the instruments are specified in Appendix A.12 [32] dealing with the power supply. Finally, relays 9 and 10 are used to deactivate the R33 of APIPy 4 and 5.

**Fig. 17:**
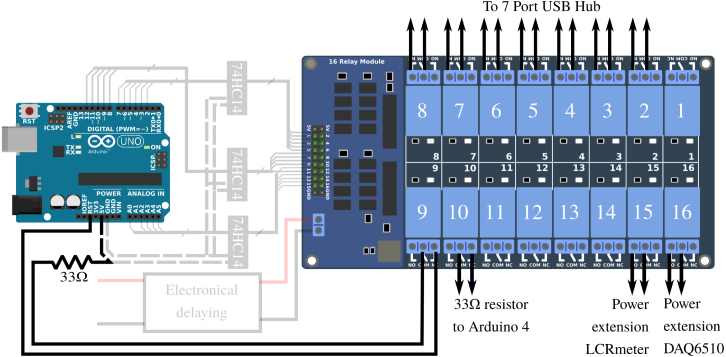
Part 2/3. Wiring map of the relays contacts of the RB16b.

All the corresponding wires are depicted in Fig. 17 are summarized in Appendix A.10 [32]. It is important to note that when thus connected, the instruments are turned off and the Arduinos are not powered when the relays are inactive, in particular when the RB16b is not powered. Furthermore, remember that the APIPy 5 is able to make itself programmable.

**Fig. 18:**
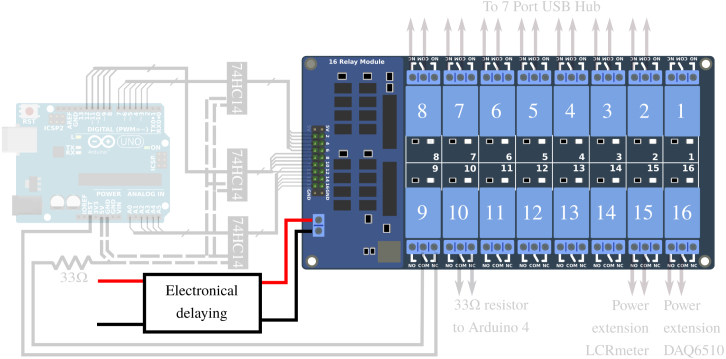
Part 3/3. Wiring map of the electronical delaying of the RB16b.

*Time delay –* The startup time constants for the relay boards and the Arduinos are different. To ensure that the RB16b comes on well after the APIPy 5, we chose to install a simple electronic time delay on the RB16b +5V power supply (Appendix A.11 [32]) (see Fig. 18).

### 5.6 Deployment of the system

In the descriptions above, all the information concerning the equipment used and the electrical connections have been presented. The addition of all these electronic sub-systems to the refrigerator, the main structure, leaves room for many design options. Hereafter, we will present the choices we made for the installation of the system.

**Fig. 19:**
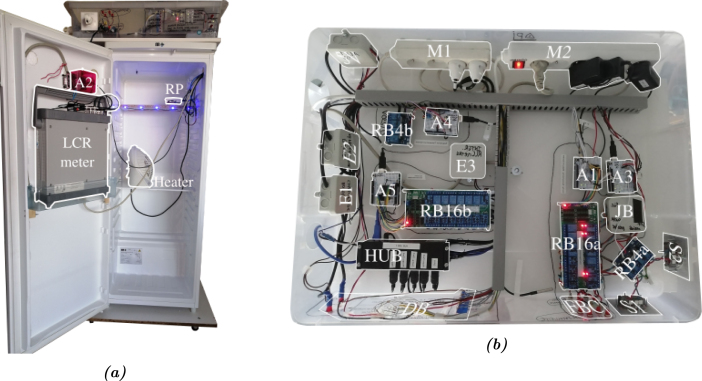
(a) Picture of the facility installed inside the casemat. The C and D cables from the terminal box are connected to the domestic sockets (top left). The LCRmeter is in the fridge. The picture was taken before the installation of the DAQ which is now in the fridge. (b) Top view of the electronical devices installed out of the fridge. Almost all the PentaPus is installed in a transparent plastic box, allowing direct visual access and easy handling. A dashboard is present in the front panel of the box but its wiring is not presented in this article. The power extension of the DAQ is also not present.

*Installation –* We decided to place certain sensitive devices inside the refrigerator, to protect them. These were two measuring instruments and the Raspberry. The external hard drive and the APIPy 2 were also installed inside the refrigerator (Appendix B.3 [32]). The rest of our devices were set up outside the refrigerator, in a transparent plastic box. In this plastic box, there are the APIPy Arduinos mounted on their prototyping boards, the four relay boards, the USB hub, the power supply buses, the extension of the power supply cables enabling their modifications, and the junction box for the external ends of the loop. Fig. 19 and Appendix A.14 [32] give a complete description of this installation.

In particular, Fig. 19 gives a detailed view of how the electrical distribution of the measurement bench is set up. Multi-sockets are installed in the plastic box. Busbar trunking systems enable the cables to be well positioned. An opening was also made to let the cables through. Furthermore, the layout of the different items in the box was designed in order to separate the part of the setup where the loop’s analogue signal arrives from the digital systems.

*Integration of the device between the loop and the instruments –* The two pairs of ends (int and ext) from the two sub-loops are each linked to a household electrical plug. Installing this household electrical socket on the front of the box and opening to the outside enabled the loop to be easily connected and disconnected from the system within. Behind the socket and plug, cables corresponding to the four ends are connected to the RB4a. Once routed by the RB16a, the electrical signal of interest is made available on the front of the plastic box by simple banana-type terminals.

*Overall summary –* It is important to note that the theoretical number of configurations for the state of each of the forty relays is huge. Among them, only a limited number correspond to a useful electrical state. These states are the only reachable states and they correspond to:

• The loop is connected to the Earth.
• The loop is connected to the LCRmeter into 11-turn series configuration.
• The loop is connected to the DAQ6510 into 11-turn series configuration.
• The loop is connected to the measuring instrument power supply into 11-turn series configuration.
• The loop is connected to the feedback giant fluxmeter into 5/6-turn series configuration.

As well as these possibilities, there are those related to the management of the power supply to the instruments and to the 33 Ω resistor piloting, independent from the list above. These different states are completely managed by the PentaPus architecture and are naturally translated in the Arduinos’ code structure (Appendix B.1 [32]).

We chose to include LEDs that serve as indicator lights showing the real-time state of the system. Their presence is not necessary during operation as the bench is piloted remotely, but they have been very useful during the development. They may be polarized with the GPIOs available on the different Arduinos and by adding the corresponding commands in the correct “switch cases” of the scripts .ino. They can also be directly connected to the different inverter units. To simplify the text, the LED connections are not presented in this article.

## 6 Operation instructions

This section is divided into 4 sub-sections. After the definition of an initial state in sub-section 1, user instructions based on two relevant scenarios are given in sub-sections 2 and 3. Finally, some advanced functionalities are presented and illustrated in sub-section 4. Here the Raspberry and the Arduinos are considered to be configured as described in Appendix A.13 [32]. It is assumed that the users know the basics for on-line commands via the SSH protocol, as well as the basics for Linux command lines. In particular, they are supposed to know how to navigate easily in the tree, execute a program in the background with the nohup command, execute a Python script, upload a file to an Arduino, and edit a text file.

### 6.1 Initial state

Before the PentaPus is powered up, the relay boards have no power and their relays are inactive. As seen in the wiring presented in the Section 5.3, when in this state, the loop is earthed and the measuring instruments are turned off. During the PentaPus power-up, the Raspberry starts up and the Arduinos are powered. Their scripts are planned not to change anything in the relay states. APIPy 4 automatically starts up the temperature regulation. This overall state of the PentaPus, of the loop, and of the measurement devices is called a standby state.

### 6.2 LCRmeter measurement

The principle of measurement by the LCRmeter involves reproducing the same measurement, regularly in time. The Python script is therefore based on a loop enabling the repetition of the same measurement. The total number of measurements is set by the user directly in the parameter file. At the end of the measurement process, the loop is re-earthed.

Measurement by the LCRmeter requires a sequence of four operations. The first involves configuring the loop’s electrical contacts. The second turns in the LCRmeter and routes the signal from the loop to its input terminals. Settings for the measurement must be entered during the third step. Finally the fourth step is to command the measurement to begin. In this sub-section, details are given about the commands necessary for measurement execution. The PentaPus response is not included, to simplify reading this article. However, each Python script will answer the user each time it is executed, via returns to the terminal.

The loop configuration is obtained by two commands. The first one disconnects the loop from the earth, to connect it to the measurement bench. In the directory 01_EarthingConnection (Code 4 of Appendix B.2 [32]), enter the command:

**Figure:**
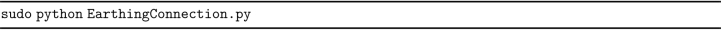

The second one enables the loop to be configured in 11 wires in series. Enter the command:

**Figure:**
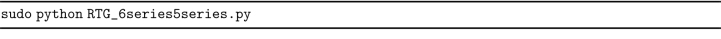

To route the electrical signal to the LCRmeter, use the directory 02_Routing (Code 4 of Appendix B.2 [32]) and enter the command:

**Figure:**
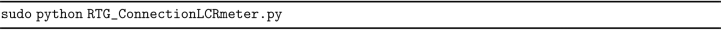

The moment when the LCRmeter is turned on is not critical. This operation must just be done before executing the measurement script. To do so, in the directory 06_Reset (Code 4 of Appendix B.2 [32]), enter:

**Figure:**
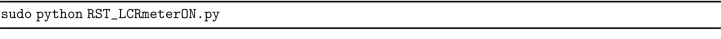

The device is relatively long to start up (about a minute), and so the script waits a few moments before re-enabling the user.

The settings for the measurement are entered in a text file, non-formatted, and read as an input file by the Python script. To edit this file in command line, the user can implement any text editor. The native editor nano is perfectly well-adapted to remote implementation. Edit the measurement settings file via the command in the directory 03_LCRmeterGPIB (Code 4 of Appendix B.2 [32]):

**Figure:**
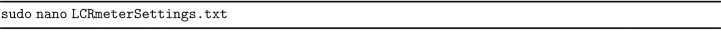

When the settings have been entered, the measurement script must be executed in a background task mode, enabling the Python procedure to continue even if the user disconnects from the Raspberry. To do this, use the command nohup (Appendix B.5). In this case, it is essential to finish the command line with the symbol &. Under Linux, this symbol enables the user to take back control on the console after execution of a command:

**Figure:**
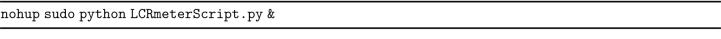

### 6.3 DAQ measurement

It is necessary to turn off the LCRmeter before using the multimeter (see above), as these two devices may influence each other when turned on. Starting from the standby state, the procedure is very similar to previous one. After entering the following commands:

**Figure:**
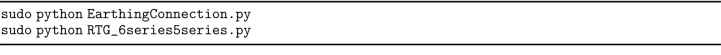

Enter:

**Figure:**
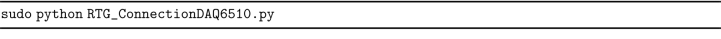

To start up the DAQ, enter:

**Figure:**
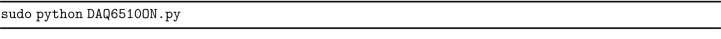

To edit the configuration file for the DAQ measurement in the directory 04_DAQ6510 (Code 4 of Appendix B.2 [32]), enter:

**Figure:**
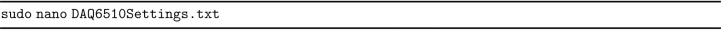

Then to launch to measurement process (Appendix B.6), use the same directory and enter:

**Figure:**
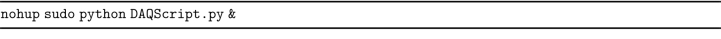

### 6.4 Advanced functionalities

Thanks to APIPy 5, it is possible to remotely disconnect the APIPys or the USB link between the DAQ and the Raspberry and also to reprogram it. Finally, APIPy 4 allows to record temperature. To do so, several advanced functionalities are carried out.

*Turning off the APIPys and the DAQ USB port –* For each APIPy, a Python script allows its restarting. These scripts are based on three phases of functioning. The first one involves opening the USB port relays of the APIPy to be restarted, the second gives a waiting time of a few seconds, and in the third the relay is closed again. The procedure is similar to disconnect all these USB links, and so we give here an example for restarting the APIPy2. Enter the command:

**Figure:**
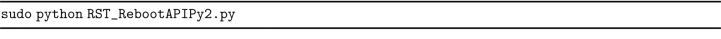

To disconnect an APIPy for a long period, just execute the first phase of the script, and to restart it, just the third one. To do this, users can create Python scripts commenting the codes that are not useful for their application.

*Reprogramming APIPy –* PentaPus is able to allow the remote reprogramming of the two APIPy4 and APIPy5 tentacles. To do so, the 33 Ω resistor installed in order to prevent a random re-initialization must be disconnected (Appendix A.1 [32]). As the two procedures are similar, we will take the APIPy4 reprogramming as an example. Enter:

**Figure:**
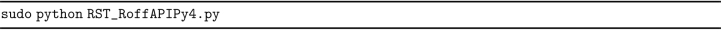

In the directory containing the new sources .ino of APIPy4, launch the Arduino programming graphic interface. Enter:

**Figure:**
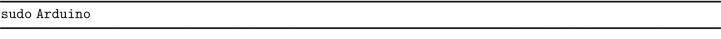

It is then necessary identify the target Arduino in the graphic environment. To do so, it is very useful to have first defined the symbolic links that allow a chosen name to be given to each Arduino (see Appendix B.4 [32]). Supposing this step has been carried out, use the command console to enter:

**Figure:**
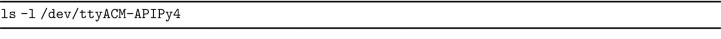

The console response is:

**Figure:**
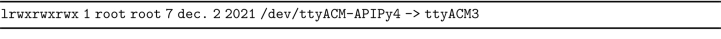

Thus the Arduino displayed as ttyACM3 on the Arduino graphic interface is indeed the APIPy4. Next, the new source just has to be uploaded to ttyACM3 via the Arduino graphic interface.

After the reprogrammation, the 33 Ω resistor is reactivated using the following command:

**Figure:**
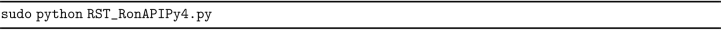

*Temperature recording –* APIPy4 regulates the refrigerator temperature as soon as it is started up. This option is not automatic, and needs to be ordered. To do so, enter the command:

**Figure:**
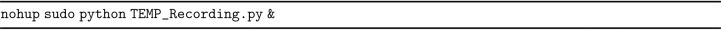

This Python script can absolutely run at the same time as the main Python scripts dedicated to electrical measurement. To stop the recording, the command kill must be used on the process identifier, obtained via the command ps -ef — grep python.

## 7 Validation and first characterization

Each APIPy was checked before the complete installation on the LSBB site. Because the APIPys 1, 2, 3, and 5 are directly involved in the measurement process, the best proof that they are working well lies in the reliability, reproducibility and longevity of each kind of electrical measurement. The well functioning of the APIPy 4 can be checked independently. This section present an in-situ validation of all these electronic devices in 3 sub-sections dedicated respectively to the temperature regulation, to the active measurements using the LCRmeter and to the passive measurements unique the DAQ. A last sub-section characterize the influence of the PentaPus on electrical measurements. Finally, a standalone test of the measuring bench was carried out to ensure that it was working properly, independently of the connection to the loop. We also closely monitored the temperature control. Details of the functional test and temperature monitoring are given in Appendix C [32].

### 7.1 Validation of active measurements

*Principles of measurement –* The LCRmeter carries out the complex impedance of the coil, denoted $Z_{m}$, at a given frequency, denoted $f_{m}$. The measurement principle consists in biasing the coil with a harmonic voltage and in measuring the dephasing, φ, and the amplitude of the generated current. If U and I denote the respective complex amplitude of the voltage and the current, $Z_{m}$ is defined as:

$$Z_{m}=\frac{U}{I}=\left|Z_{m}\right|\exp\left(j\varphi \right)$$

We name this functionality the “active mode”, because the coil is excited electrically by the operator. The order of magnitude of the current is around a few milliamperes, while the voltage is around a few volts.

To completely characterize the coil over a large frequency range, the LCRmeter makes the measurements at different frequencies, from typically $f_{m}=20\;\mathrm{Hz}$ up to $f_{m}=10$ kHz, with a step defined by the operator. The scanning in frequencies is called a frequency sweep. Because of the coupling between the coil and its direct environment, the result of a sweep can change with time. We therefore monitored the time evolution of $Z_{m}$ for all the frequencies $f_{m}$ of interest.

At each frequency, the measurement of $Z_{m}$ over an integration time was repeated several times, in order to obtain an average. The integration time, the number of averaged samples, the set of frequencies, and the total monitoring time are configurable by the operator using the setting file (see Section 6).

**Fig. 20:**
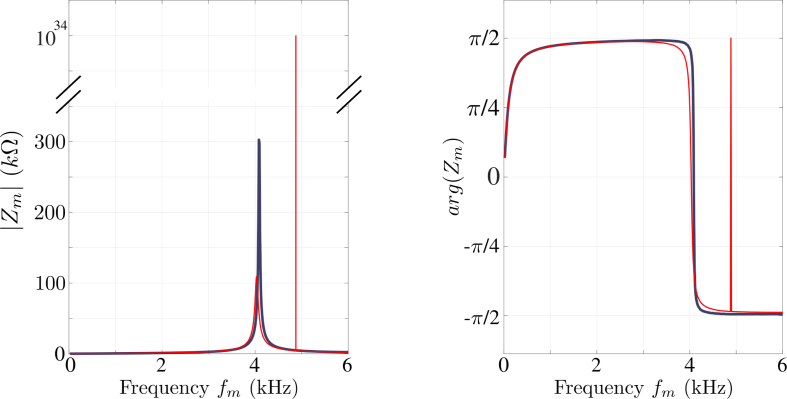
Modulus (left) and phase (right) of the complex impedance versus the measurement frequency $f_{m}$ operated 2021 February 16th at 02:00 AM (UTC) for red curves and at 05:00 AM (UTC) for gray ones. The left figure vertical axis is truncated to enlight the difference of order of magnitude. (For interpretation of the references to color in this figure legend, the reader is referred to the web version of this article.)

Typical characteristics are plotted in Fig. 20. This shows an expected resonant behavior for a coil with a maximum of $Z_{m}$ centered around $f_{m}∼4$ kHz, corresponding to a phase φ becoming null. The measurements were performed on 528 different frequencies (from 20Hz up to 6 kHz), with a long time integration (around 400 ms, depending on $f_{m}$), averaging 50 measurements at each frequency $f_{m}$. With such a configuration, each sweep has a duration of 1h30’. We bring to the reader’s attention that for each sweep, the Raspberry sends 3×528∼1600 SCPI instructions to the LCRmeter via the GPIB controller and receives in return 528 answers containing the measured values.

This figure highlights a single unpredictable measurement, around $f_{m}∼5$ kHz, with a modulus value of $10^{37}\Omega$ and a phase jump. This kind of measurement is called overload. When the LCRmeter cannot perform its measurement, it produces a number equal to the maximum it can encode. This number is around $10^{37}\Omega$ for our device. This particular behavior allows the LCRmeter to try a measurement on the next frequency and continue the sweep, as shown in Fig. 20 (red curves). The overload can occur when the amplitude calibre is too low, or when it cannot measure the phase. An overload can be produced by an occasional random malfunctioning of the measurement device (sporadic electrical problem) or an external electromagnetic event (lighting, for instance), generating a high voltage surge on the loop. Because the measurement process is not interrupted, it is therefore possible to localize the overloads in time and frequency $f_{m}$, and eventually obtaining formations about the statistics of such external phenomena.

**Fig. 21:**
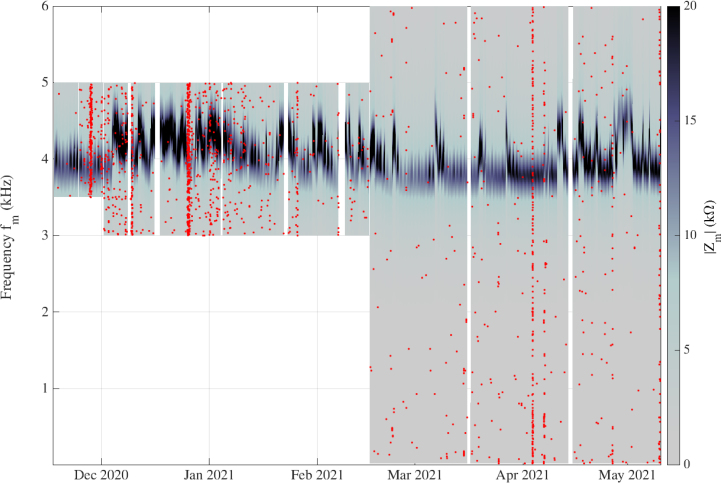
Modulus of the complex impedance (gray scale color) versus the measurement frequency $f_{m}$ (vertical axis) and the dates of measurements (horizontal axis), during six months in 2021. Each red dot indicates one overload event. For visual comfort, the size of the red dots has been chosen much greater than the resolution pixel corresponding to one measurement.

*Reliability with long-range time monitoring –* During the period from December 2020 to May 2021, the bench was configured to operate in the active mode almost non-stop. To do so, the bench had to be configured to operate a loop on the sweeps. However, during this period we had to integrate a constraint coming from a neighboring experiment. Two ultra-sensitive magnetic antennas were buried about 3 meters from the coil (outside its perimeter), in order to study atmospheric events. This device was part of a European network [6]. The two antennas were passively exploring the frequency range between 0 Hz and 1 kHz. Because the antennas were close to the coil, the active mode strongly disturbed the measurements at low frequencies (less than 1 kHz), and so from December 2020 to March 2021 our bench had to be configured to avoid this critical range of frequencies. When the two magnetic antennas were switched off after March 2021, the bench was reconfigured to measure the full frequency range of interest (20 Hz up to 6 kHz). The results obtained for the modulus of $Z_{m}$ are presented in Fig. 21, as a color map. We also plotted the overload measurements (see legend).

The data set contains $1.406784\times 10^{6}$ measurements, with $1.911\times 10^{3}$ overloads, representing 0.13%. The white rectangles on the figure correspond to the areas (time and $f_{m}$) where no measurements were performed because of the constraint imposed by the other experiment prior to March 2021, or the IT maintenance needed to relaunch the measurements at the end of the loop, or due to a power cut (unexpected or scheduled). It is important to note that all the configuration and relaunching operations were carried out remotely, without any physical intervention on the bench.

With this representation, it appears that there are two families of overloads, single events and collective events. For instance, during March 2021, overloads appeared randomly and each one was relatively far from the others. On the other hand, we can see collective events of overloads during December 2020 or April 2021, with vertical lines of red dots corresponding to a high density of overloads during one or several successive sweeps. It is also important to note that no overload stopped the sweep.

The goal of this paper is not to interpret scientific results, but to show the possibilities and the limitations of our measurement bench, so we will not develop different hypotheses as to the presence of overloads and the different ways to exploit them as information. However, we can briefly discuss this phenomenon. The density of overloads is basically linked to external electromagnetic activity, but it can be amplified by the different parameters of the LCRmeter setup. A long integration time and a large number of samples used to obtain averages increase the probability of an overload appearing during the measurement, while on the other hand drastically reducing the values of those parameters generates noisy measurements. A compromise must be reached between the different constraints and depending on the kind of measurements (low frequencies for long range phenomena such as water transport, higher frequencies for atmospheric observations, …). Because of the quite unpredictable character of overloads, and their relative infrequency, this kind of refinement will take time to be implemented. To conclude this sub-section, we highlight the reliability of the bench for three different points. In terms of hardware constraints, the bench is operational. It works in a harsh environment with its electronic devices thermally protected and protected from sunlight. No failure of an Arduino, Raspberry Pi, measurement device, or the refrigerator has occurred since December 2020. The software architecture is very satisfactory. The APIPy configuration interaction with the LCRmeter device did not produce any bugs, even with the five simultaneous connections between the Raspberry and the Arduinos. The choice of this architecture has therefore been validated, on site and a posteriori, especially because of the ease of the remote control it allows. It has been seen that the bench is quickly and remotely configurable just with the help of command lines in the Raspberry. No graphic interface (GUI) is required to enter the different parameters and order a set of measurements, or to recover the generated data. These points are very important for the overall reliability of the bench: it remains reliably accessible from everywhere with internet connection, even if the quality of bandwidth is poor.

### 7.2 Validation of passive measurements

The second mode of measurements available on the bench at this time consists in directly measuring, without any polarization, the induced voltage appearing along the coil by an induction effect. This mode is called “passive mode”. A high precision digital multimeter, often used in industrial applications, was connected directly to the coil and controlled directly by the Raspberry via a USB cable. It can be used as an acquisition data logger, since it has a memory buffer with $5.10^{6}$ points of measurements. It is possible to configure the kind of measurement, voltage or current, but it uses different physical input of the instrument. Today, this connection is not remotely controlled and the output of the PentaPus is connected to the DAQ in accordance with its voltmeter mode. Switching the multimeter from amperemeter to voltmeter would require a physical intervention on the device connections. It is however possible to configure the sampling frequency, the measurement range, and the integration time by remote connection. Because we are currently testing and validating the different possibilities of the bench, we have chosen to connect it as directly on the coil without pre-processing of the signal: no preamplifier, no filtering, no antialiasing filter. It should be noted that when the LCRmeter is switched on, the measurements produced by the multimeter are disturbed. It must therefore be turned off to run the passive mode. Because it is not recommended to start up and shut down the LCRmeter several times a day, it is not possible to automatically alternate, active and passive measurements in the same day.

**Fig. 22:**
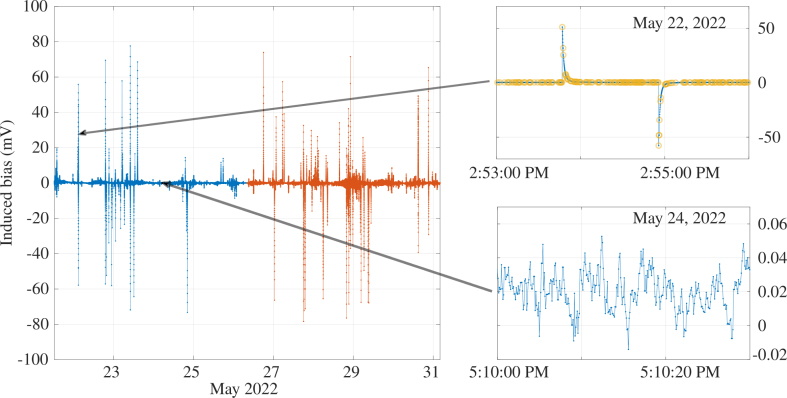
Induced voltage measured during ten days in May 2022 (22th–31st). The sampling frequency is 12Hz, the integration time is 80ms (0.96% of the period of sampling). Data set contains $10\times 10^{6}$ points, obtained during two successive records of $5.10^{6}$ points each, and separated of 4 minutes, may the 26th. Left: the totality of the chronogram, with different colors to distinguish the two records. Right up: zoom on a 3 min duration zone in may the 22nd, with overloads plotted in yellow circles. Right down: zoom on a 30 seconds duration zone the 24th during a electromagnetically quite period without any overloads.

A typical example of results provided by the multimeter is shown in Fig. 22. The configuration described in the legend was implemented remotely, as for the active mode. We have already checked the relevancy of the measurements using the disturbances generated by the electrical network at 50 Hz and its harmonics. Also in this example, it was decided to sample at 12 Hz with an integration time of each measurement equal to 80 ms (96% of the time separating two samples). The multimeter operates measurements until its buffer is full. Then it uploads the data to the Raspberry hard disk. This operation takes approximatively 4 min, during which time no measurements are made. Then it restarts, with a run of $5.10^{6}$ measurements. Fig. 22 shows two successive runs separated by this blind time window.

Using this mode, overloads appear when the measured voltage is greater than the measurement range. In this example, the number of overloads is about $8.10^{5}$ for $10^{7}$ samples, i.e. about 8%. In order to represent the signal, the overloads were processed as follows. First, the overload values ($10^{37}$) were replaced by the value of the measurement range (100 mV). Then an average was calculated with the previous and next values. In so doing, in a series of consecutive or very close overloads, the values obtained were not significant or scientifically exploitable, but the overall signal remains representable. In Fig. 22, 2 zooms are presented, one corresponding to a time window with positive and negative overloads, the other to a time window of quiet electromagnetic activity with no overloads. The first shows that this sampling frequency is not at all adapted to correct measurements of the voltage for the events generating the overload: the Shannon criterion is not respected. Moreover, the measurement range is too low for these events. The second zoom shows that the numerisation process is well adapted to this range of voltage: there is no discretization noise.

In terms of scientific exploitability of this mode, it appears the frequency range of interest must first be chosen, then a pre-treatment adapted for the signal, with a preamplifier and antialiasing filter. However, as for the active mode, the reliability of this measurement has been demonstrated, since it is operational, and can be completely remote controlled. The switch between the two modes can be operated remotely.

## 8 Perspectives and conclusion

Detailed explanations concerning the hardware and software developments of a device based on well-adapted and open-source technologies and dedicated to the instrumentation of a giant loop are given in this article. The whole device is original not only because of the extraordinary size of the loop and of its location in a unique scientific site but also because of the type of measurement produced. Validation was obtained as to the correct functioning of the two types of measurements implemented. The architecture of the developed device was based on the association of Arduinos, Raspberry, and Python, named APIPy, deployed in the form of a five-tentacle “octopus” named PentaPus and piloting a set of relay boards as well as the measurement devices. The robustness and reliability of the developed system have been proven over a suitably long period of time. To date, the system has continued to operate and is still taking measurements. This prototype needed to be versatile, as it is used in research activities that intrinsically are never static. The use of relay boards has proven to be perfectly suited to a simple, practical, and reliable deployment.

Three broad families of perspectives open up following the work already accomplished. The first concerns the technical development of this measurement bench. One evolution that could be envisaged is to integrate the PentaPus on a system of electronic boards designed in order to optimize the number of connections and the size, and making the addition of measurement devices even easier. This evolution, though interesting from a technical point of view is, however, not a scientific priority as long as the present system functions well and does not require major changes. In fact, there are still two free places on the bench, and the GPIB bus would allow the addition of instruments in cascade on the LCRmeter. The second family of future possibilities concerns the production of exploitable scientific results. In particular, the use of the passive mode will require the implementation of an anti-aliasing filter, optimization of the sampling frequencies, and a signal processing procedure that would enable this data to be cross-referenced with the multiphysics measurements carried out on the site. These could include gravimetric monitoring, meteorological monitoring, electromagnetic monitoring with different types of antennas, or even evapo-transpiration measurements of the site’s vegetation. Furthermore, the use of a low noise trans-impedance amplification system would be required. In the longer term, we will need to deploy a magnetic flux measurement system. In parallel with these actions, it will be necessary to train the different scientific communities in the use of this measurement tool. One of the commitments among our objectives is that it should be shared.

The third and final family of perspectives involves open-source technology transfer and the sharing of our know-how. Already, the long-term deployment of smaller magnetic sensors on agricultural land can generate interest. More generally, the robust, low-cost, and open-source architecture of PentaPus opens up the possibility of positioning measurement systems in harsh environments more confidently than would be allowed by expensive proprietary boards, which furthermore become obsolete rapidly. One of the declared goals of this article is to share our experience in order to encourage inspiration in the domain of piloting complex electronic systems in extreme environments.

## CRediT authorship contribution statement

**Clément Dezord:** Conceptualization, Methodology, Software, Writing – original draft. **Gilles Micolau:** Methodology, Validation, Supervision, Writing – review & editing. **Chahine Abbas:** Writing – original draft, Formal analysis. **Arnaud Mesgouez:** Supervision, Writing – review & editing. **Elisabeth Pozzo Di Borgo:** Supervision.

## Declaration of competing interest

The authors declare that they have no known competing financial interests or personal relationships that could have appeared to influence the work reported in this paper.
